# Structural transformation and catalytic hydrogenation activity of amidinate-protected copper hydride clusters

**DOI:** 10.1038/s41467-022-29819-y

**Published:** 2022-04-19

**Authors:** Chun-Yu Liu, Shang-Fu Yuan, Song Wang, Zong-Jie Guan, De-en Jiang, Quan-Ming Wang

**Affiliations:** 1 Department of Chemistry, Key Laboratory of Organic Optoelectronics and Molecular Engineering of the Ministry of Education, Tsinghua University, 10084 Beijing, PR China; 2 Department of Chemistry, University of California, Riverside, CA 92521 USA

**Keywords:** Ligands, Catalytic mechanisms, Heterogeneous catalysis

## Abstract

Copper hydrides are important hydrogenation catalysts, but their poor stability hinders the practical applications. Ligand engineering is an effective strategy to tackle this issue. An amidinate ligand, N,N′-Di(5-trifluoromethyl-2-pyridyl)formamidinate (Tf-dpf) with four N-donors has been applied as a protecting agent in the synthesis of stable copper hydride clusters: Cu_11_H_3_(Tf-dpf)_6_(OAc)_2_ (**Cu**_**11**_) with three interfacial *μ*_5_-H and [Cu_12_H_3_(Tf-dpf)_6_(OAc)_2_]·OAc (**Cu**_**12**_) with three interstitial *μ*_6_-H. A solvent-triggered reversible interconversion between **Cu**_**11**_ and **Cu**_**12**_ has been observed thanks to the flexibility of Tf-dpf. **Cu**_**11**_ shows high activity in the reduction of 4-nitrophenol to 4-aminophenol, while **Cu**_**12**_ displays very low activity. Deuteration experiments prove that the type of hydride is the key in dictating the catalytic activity, for the interfacial *μ*_5_-H species in **Cu**_**11**_ are involved in the catalytic cycle whereas the interstitial *μ*_6_-H species in **Cu**_**12**_ are not. This work highlights the role of hydrides with regard to catalytic hydrogenation activity.

## Abstract (web summary)

Copper hydrides have been studied for their exciting structural chemistry and applications in hydrogenation catalysis. Here, the authors uncover the role of the amidinate ligand in yielding two closely related copper hydride clusters with quite different catalytic hydrogenation activity.

## Introduction

Copper hydrides have historically been studied for their exciting structural chemistry and applications in hydrogenation catalysis and hydrogen-storage technology^1–7^. Recently, intense attention has been paid to synthesize atomically precise copper hydride clusters. A series of copper hydride clusters with bidentate ligands have been reported, which contain bridging (*μ*-H), capping (*μ*_3_-H) and interstitial (*μ*_(4−6)_-H) hydrides^8–16^. The precise control over number of hydrides and their arrangements within these copper hydride clusters could provide valuable possibilities in modulating their catalytic hydrogenation activity. However, these copper hydride clusters are usually not stable enough and lose their identity quickly in solution^17–19^, which presented synthetic difficulties and limited their wide application.

Surface organic ligands are critical in the construction and stabilization of atomically precise metal nanoclusters^20–27^, ligand engineering is an important approach in promoting the stability of copper hydrides. Envisioning multidentate amine ligands could provide stronger protection to metal clusters due to their multiple binding sites and their anionic nature which is helpful for ligating cationic metal ions^28–32^, we chose an amidinate ligand, N,N′-Di(5-trifluoromethyl-2-pyridyl)formamidinate (Tf-dpf) containing four N-donors, as the protecting agent for copper hydride clusters. Such a strong protection of ligand shell favors the high stability of copper hydride clusters. Moreover, Tf-dpf has a flexible linear structure favoring the generation of metal cluster diversity^30^, which may be constructive in establishing structure-property relationships in terms of hydrogenation catalysis.

**Fig. 1:**
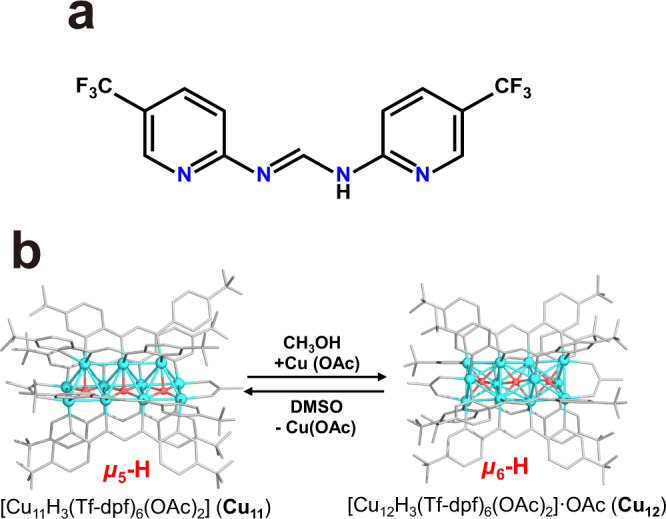
The structure of Tf-dpf ligand and the solvent-induced interconversion of Cu_11_ and Cu_12_. The chemical structure of Tf-dpf (**a**) and the structural transformation between **Cu**_**11**_ and **Cu**_**12**_ (**b**). Color legend of **b**: light blue, Cu; red, Hydride; white, Tf-dpf ligand.

Herein, we report two amidinate-protected copper hydride clusters Cu_11_H_3_(Tf-dpf)_6_(OAc)_2_ (**Cu**_**11**_) and [Cu_12_H_3_(Tf-dpf)_6_(OAc)_2_]·OAc (**Cu**_**12**_), and their reversible interconversion (Fig. 1). The hydride positions in **Cu**_**11**_ and **Cu**_**12**_ were further confirmed by a machine-learning model based on convolutional neural networks (CNN) and trained on published structures of copper hydride clusters from neutron diffraction. It is quite unexpected that **Cu**_**11**_ showed high activity in the reduction of 4-nitrophenol (4-NP) to 4-aminophenol (4-AP), while **Cu**_**12**_ displayed very low activity. Structural determination of these two clusters revealed that the type of hydride is the key in dictating the catalytic activity. **Cu**_**11**_ has three interfacial *μ*_5_-H and **Cu**_**12**_ has three interstitial *μ*_6_-H. Deuterated catalytic experiments confirmed that the *μ*_5_-H of **Cu**_**11**_ is involved in the catalytic cycle whereas the *μ*_6_-H of **Cu**_**12**_ is not active_._ These findings are not only helpful for understanding the catalytic mechanism, but also instructive for the design and synthesis of efficient hydrogenation catalysts.

## Results

### Synthesis and characterization

HTf-dpf ligand was synthesized by heating the mixture of 5-(trifluoromethyl)-2-aminopyridine and excess triethyl orthoformate (TEOF) at 120 °C under nitrogen atmosphere (Supplementary Fig. 1)^33^. The preparation of **Cu**_**12**_ involves the direct reduction of a mixture of Cu(OAc) and HTf-dpf/Et_3_N with a mild reducing agent, Ph_2_SiH_2_ in a mixed CH_2_Cl_2_/CH_3_OH solvent. **Cu**_**11**_ was obtained by changing the reaction solvent to CH_2_Cl_2_/DMSO (dimethylsulfoxide), and then crystallized from CH_2_Cl_2_ and n-hexane. ^1^H NMR spectroscopic analysis of **Cu**_**11**_ (Supplementary Fig. 2a) and **Cu**_**12**_ (Supplementary Fig. 3a) in CD_3_OD identified four sets of aromatic resonances corresponding to Tf-dpf ligands. Three OAc^−^ in **Cu**_**12**_ are divided into two groups in a 2:1 ratio based on their environments. Three hydrides in **Cu**_**11**_ gave ^1^H NMR signals at 2.34 (2H) and 3.15 (1H) ppm, and similar ^1^H NMR shifts were found at 1.46–2.80 ppm for [Cu_20_H_11_(S_2_P(OiPr)_2_)_9_]^17^, and 2.18-3.44 ppm for [Cu_29_Cl_4_H_22_(Ph_2_phen)_12_]Cl (Ph_2_phen = 4, 7-diphenyl-1,10-phenanthroline)^34^. The observed values of **Cu**_**12**_ at 5.64 (2H) and 7.16 (1H) ppm are comparable to those in Cu_28_H_16_(dppe)_4_((4-isopropyl)thiophenol)_4_(CH_3_CO_2_)_6_Cl_2_ at 3.4-6.3 ppm^35^, and the encapsulated hydrides in [Cu_8_H{S_2_CC(CN)_2_}_6_]^5−^ and [Cu_8_H{S_2_C(NEt_2_)}_6_]^−^ at 7.02 and 7.6 ppm^36,37^, respectively. In addition, ^19^F NMR of **Cu**_**11**_ (Supplementary Fig. 2b) and **Cu**_**12**_ (Supplementary Fig. 3b) in CD_3_OD show one singlet at −63.28 and −63.88 ppm, respectively (free HTf-dpf presents at −60.21 ppm, Supplementary Fig. 1b), suggesting that the six Tf-dpf ligands in **Cu**_**11**_ and **Cu**_**12**_ are in similar environments, respectively.

**Fig. 2:**
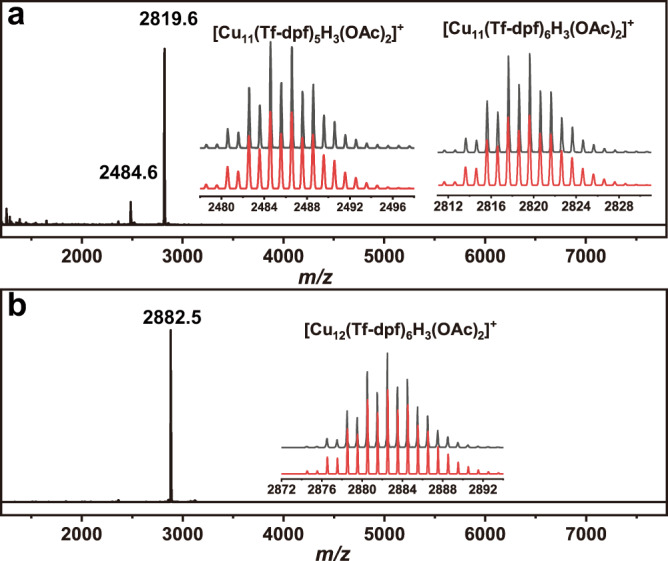
ESI-MS of Cu_11_ and Cu_12_ in MeOH. Mass spectra of **Cu**_**11**_ (**a**) and **Cu**_**12**_ (**b**), inset: the measured (black trace) and simulated (red trace) isotopic distribution patterns of the corresponding the molecular ion peaks.

As shown in Fig. 2a, the positive ESI-MS spectrum of **Cu**_**11**_ shows two prominent peaks, corresponding to the molecular ion [Cu_11_H_3_(Tf-dpf)_6_(OAc)_2_]^+^ (*m*/*z* = 2819.64) and [Cu_11_H_3_(Tf-dpf)_5_(OAc)_2_]^+^ (*m*/*z* = 2484.56). The spectrum of **Cu**_**12**_ gave signal of molecular ion [Cu_12_H_3_(Tf-dpf)_6_(OAc)_2_]^+^ at *m*/*z* = 2882.51 (Fig. 2b). The observed isotopic patterns of the clusters are in perfect agreement with the simulated. UV–vis absorption spectra of **Cu**_**11**_ and **Cu**_**12**_ in MeOH display three prominent absorption bands at 238, 288, and 340 nm, which are corresponding to the intraligand transitions of the Tf-dpf ligand, as similar bands are found in HTf-dpf (Supplementary Fig. 4).

To our surprise, **Cu**_**11**_ and **Cu**_**12**_ are very stable under ambient conditions. In the solid state, they are air and moisture stable (Supplementary Fig. 5). In addition, **Cu**_**11**_ and **Cu**_**12**_ are stable in solution (even in polar solvents such as CH_2_Cl_2_) for 2 weeks (Supplementary Fig. 6).

### Molecular structures

**Fig. 3:**
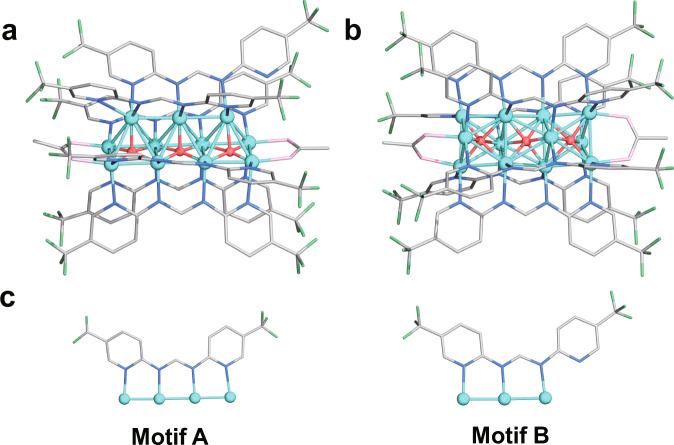
Molecular structures of Cu_11_ and Cu_12_. Total structure of Cu_11_H_3_(Tf-dpf)_6_(OAc)_2_ (**a**) and [Cu_12_H_3_(Tf-dpf)_6_(OAc)_2_]^+^ (**b**). Schematic representation of the binding modes of Tf-dpf in **Cu**_**11**_ and **Cu**_**12**_ (**c**). Color legend: light blue, Cu; green, F; blue, N; pink, O; gray, C; red, Hydride.

Single-crystal X-ray diffraction (SCXRD) structural analysis (Supplementary Table 1) revealed that **Cu**_**11**_ comprises a Cu_11_H_3_(Tf-dpf)_6_(OAc)_2_ cluster (Fig. 3a and Supplementary Fig. 7), wherein six Tf-dpf ligands are ligated to Cu_11_(*μ*_5_-H)_3_ core in a linear pattern (four in motif A and two in motif B) with Cu–N bond lengths ranging from 2.026(4) to 2.131(4) Å (Supplementary Table 2). Two OAc^−^ anions bind the two copper atoms at the ends of the linear Cu_11_(*μ*_5_-H)_3_ unit, giving the Cu−O bond lengths of 2.021(4) to 2.038(4) Å. The metal core of **Cu**_**11**_ could be regarded as the fusion of three edge-sharing rectangular pyramids. The Cu…Cu distances of the **Cu**_**11**_ skeleton range from 2.428(1) to 2.749(1) Å.

The structure of **Cu**_**12**_ includes a [Cu_12_H_3_(Tf-dpf)_6_(OAc)_2_]^+^ cationic cluster (Fig. 3b) and a OAc^−^ counter anion (Supplementary Fig. 8). The coordination modes of OAc^−^ in **Cu**_**12**_ are similar to that of **Cu**_**11**_, with the Cu–O bond lengths ranging from 2.111(5) to 2.120(4) Å. The six Tf-dpf ligands in **Cu**_**12**_ adopt distorted motif A binding mode, with the Cu–N bond ranging from 2.000(5)–2.096(5) Å. The metal core of **Cu**_**12**_ could be regarded as the fusion of three face-sharing octahedra. Moreover, the 12 copper atoms in **Cu**_**12**_ are typical hexagonal close-packed type structure with ABAB packing mode (Supplementary Fig. 9). Cu…Cu distances of **Cu**_**12**_ skeleton range from 2.497(1)–2.764(1) Å, which is much longer than that of **Cu**_**11**_. Shorter Cu…Cu contact in **Cu**_**11**_ could be attributed to the linear coordination mode of Tf-dpf, while Tf-dpf adopts zigzag coordination mode in **Cu**_**12**_ to form relatively longer Cu…Cu contacts (average 2.657 Å) as shown in Fig. 3c. These Cu…Cu distances observed in **Cu**_**12**_ is comparable to the average Cu…Cu contact of 2.66 Å in the Cu_6_ octahedral structures^38^.

### Neural network prediction of hydride sites

**Fig. 4:**
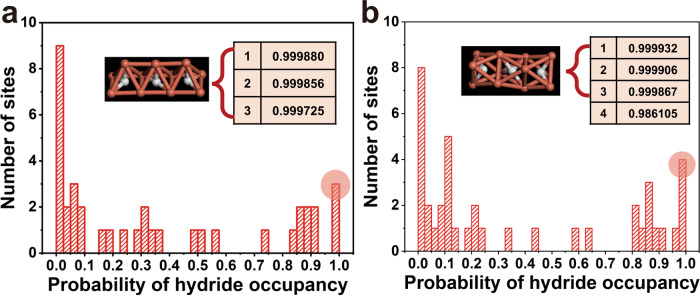
Neural network prediction of hydride sites. Probability distribution of hydride occupancy in the possible sites as predicted by convolutional neural network: **a** **Cu**_**11**_H_3_; **b** **Cu**_**12**_H_3_. Insets show the probabilities of the top-ranked sites and their positions in the clusters before DFT optimization. Color legend: Cu, dark salmon; Hydride, white.

Even though the location of H atoms by SCXRD is difficult, the hydrides in **Cu**_**11**_ and **Cu**_**12**_ could be estimated based on the charge distribution in their cluster frameworks and refined freely. Although attempts to grow single crystals suitable for neutron diffraction were unsuccessful, we applied a recently developed machine-learning model based on CNN to confirm the hydride location. The CNN method can quickly predict hydride occupancy in a Cu cluster given the heavy-atom coordinates^39,40^. We fed the SCXRD-determined positions of heavy-atoms into the CNN model and predicted the most probable sites in the two clusters. As shown in Fig. 4, the CNN model predicted close-to-1 occupancies in three sites for both the **Cu**_**11**_ and **Cu**_**12**_ clusters. The locations of these top three sites are shown in Fig. 4 insets; indeed, they exactly match the sites determined from SCXRD. Further density functional theory (DFT) geometry optimizations confirmed the stability of these clusters, as the cluster framework was well maintained after structural relaxation with hydrides at the predicted sites, and the SCXRD and DFT structures were in good agreement (Supplementary Table 2). For the sites with probability around 0.7–0.9, we would normally consider them as well, but the **Cu**_**11**_ and **Cu**_**12**_ clusters are much smaller and their structures are much simpler, quite resembling the structures in our training set. So the most probable three sites from our machine-learning model happen to be the most viable model that agrees with the SCXRD and is further confirmed by DFT.

In **Cu**_**11**_, three hydrides are disposed in the center of Cu_4_ square with an approximate square pyramidal *μ*_5_ coordination mode. Of note, the positions of the three *μ*_5_-H in **Cu**_**11**_ relative to the centers of Cu_4_ squares differ slightly: the middle one is right in the center of Cu_4_ square and the other two are deviated from the centers of Cu_4_ squares (close to the OAc^−^). The Cu-H distances were found in the range from 1.61(6) to 2.02(7) Å. In **Cu**_**12**_, three hydrides are disposed in the center of Cu_6_ octahedra with a *μ*_6_-H coordination mode. Similar to that in **Cu**_**11**_, the middle H was found to be right in the center of Cu_6_ octahedron, while the other two show offsets closing to the OAc^−^. The Cu-*μ*_6_-H distance in **Cu**_**12**_ ranges from 1.71(8) to 2.02(6) Å.

### Interconversion

**Fig. 5:**
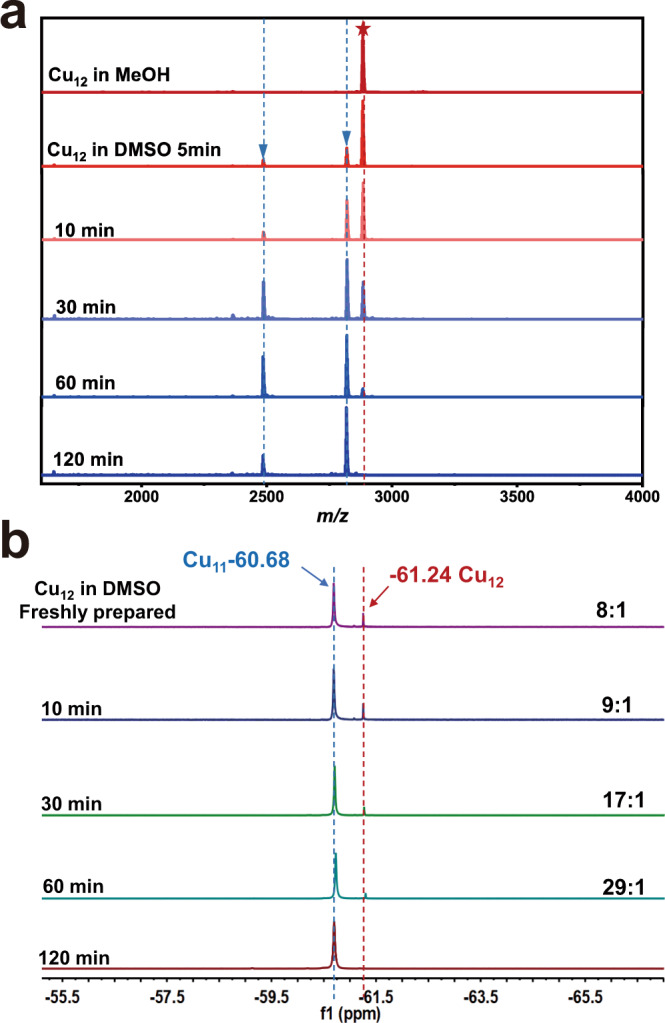
Conversion of Cu_12_ to Cu_11_. **a** Changes in the ESI-MS spectrum of **Cu**_**12**_ in DMSO. **b** Changes in the ^19^F NMR spectrum of **Cu**_**12**_ in DMSO-*d*_6_.

Interestingly, it was found that the interconversion between **Cu**_**11**_ and **Cu**_**12**_ could be triggered by solvents. The interconversion involves the adding a Cu^+^ ion to **Cu**_**11**_ or leaving of a Cu^+^ ion from **Cu**_**12**_. Dissolving **Cu**_**12**_ in DMSO led to the leaving of a Cu^+^ ion to form **Cu**_**11**_, while the reaction of **Cu**_**11**_ with CuOAc (1 equiv) in CH_3_OH converted it back to **Cu**_**12**_. The interconversion was not affected by O_2_ for the same interconversion was observed both in the air and under nitrogen atmosphere. The flexible arrangement of the N donors of Tf-dpf makes such an interconversion possible, which allows keeping stable ligation to metal ions in adjusting to the structural changes between **Cu**_**11**_ and **Cu**_**12**_. To better understand the cluster-to-cluster transformation process, we monitored the cluster core transformation process (**Cu**_**12**_ to **Cu**_**11**_) by ESI-MS measurements (Fig. 5a). The MeOH solution of **Cu**_**12**_ features one prominent peak attributed to [Cu_12_H_3_(Tf-dpf)_6_(OAc)_2_]^+^. The freshly prepared **Cu**_**12**_ solution in DMSO showed peaks corresponding to [Cu_11_H_3_(Tf-dpf)_6_(OAc)_2_]^+^ along with a weak peak attributed to [Cu_11_H_3_(Tf-dpf)_5_(OAc)_2_]^+^ within 5 min. Then the peaks of **Cu**_**11**_ keep increasing with time, and after 120 min the spectrum features only prominent peaks of **Cu**_**11**_ while the peak of **Cu**_**12**_ disappeared, indicating the complete conversion from **Cu**_**12**_ to **Cu**_**11**_.

We then monitored a solution of **Cu**_**12**_ in DMSO-*d*_6_ at room temperature by measuring its ^19^F NMR spectra at different times. A slight upfield shift of ∼2.6 ppm of **Cu**_**12**_ and **Cu**_**11**_ was found in DMSO-*d*_6_ compared with in CD_3_OD. As shown in Fig. 5b, the signal at −61.24 ppm of **Cu**_**12**_ gradually disappeared, while that at −60.68 ppm of **Cu**_**11**_ gradually grew with increasing time, indicating a transformation of **Cu**_**12**_ to **Cu**_**11**_ in DMSO at room temperature. Based on the integration ratios relative to the internal standard, the conversion of **Cu**_**12**_ to **Cu**_**11**_ is virtually quantitative.

**Fig. 6:**
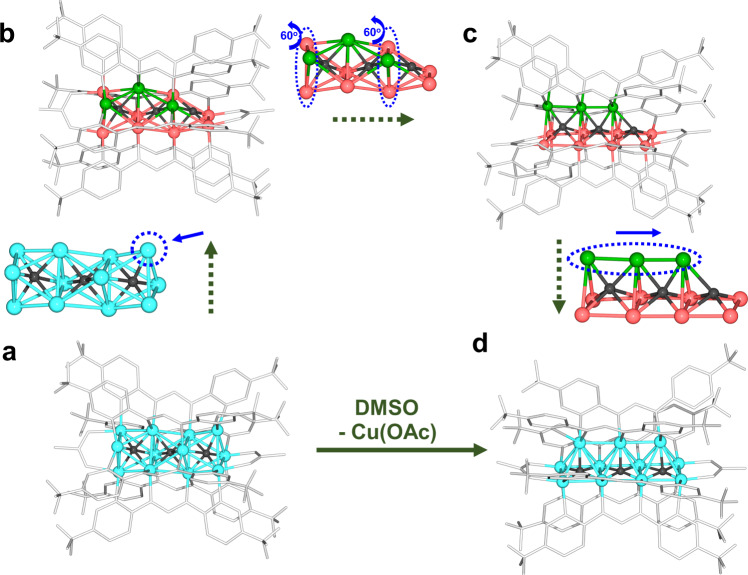
Hypothetic step-by-step transformation from Cu_12_ to Cu_11_. **a**, **b** DMSO takes away an unsaturated copper atom; **b**, **c** two Cu_3_ units of **Cu**_**12**_ twist for 60° along with the binding mode of Tf-dpf changes; **c**, **d** the translation of linear Cu_3_ unit. Color legend: light blue, red, and green Cu; gray, Tf-dpf; black, Hydride.

Given the different numbers of Cu atoms in **Cu**_**11**_ and **Cu**_**12**_, the transformation between the two clusters is not isomerization. As shown in (Supplementary Fig. 10), the OAc^−^ only binds two out of the three terminal copper atoms of the **Cu**_**12**_ core, and the other one copper atom could be regarded as an unsaturated site. Thus, it is hypothesized that the transformation of **Cu**_**12**_ to **Cu**_**11**_ is attributed to the binding ability of DMSO, which anchors on the unsaturated copper atom and removes it from the cluster. As a result, the binding mode of Tf-dpf in **Cu**_**12**_ is distorted motif A, which leads to the twisting of two Cu_3_ units and then the framework rearrangement to form **Cu**_**11**_ (Fig. 6). Moreover, the conversion of **Cu**_**11**_ to **Cu**_**12**_ through adding CuOAc in CH_3_OH proves that **Cu**_**11**_ is likely to combine free Cu ions to generate **Cu**_**12**_ (Supplementary Fig. 11).

### Hydrogenation catalysis

Synthesis of anilines or amines from the corresponding nitro compounds is an important process in both of the laboratory and the chemical industry due to their versatility in several biologically active natural products, pharmaceuticals, and dyes^41^. Transition metal-catalyzed hydrogenation is an important route for the transformation of nitro groups to amine groups^42,43^. Thus, the reduction of 4-NP to 4-AP by NaBH_4_ was chosen as a model reaction to investigate the catalytic performance of **Cu**_**11**_ and **Cu**_**12**_. Considering that **Cu**_**11**_ and **Cu**_**12**_ are insoluble in water, this catalytic reaction belongs to heterogeneous catalysis.

**Fig. 7:**
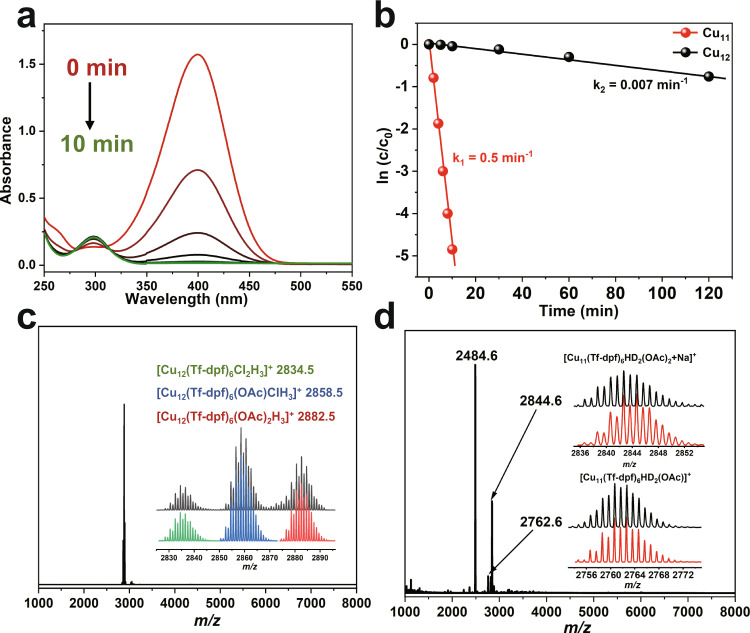
Catalytic performance of Cu_11_ and Cu_12_. **a** UV–Vis spectra showing gradual reduction of 4-NP catalyzed by **Cu**_**11**_. **b** Plot of −ln(c/c_0_) vs. reaction time during the reduction of 4-NP with **Cu**_**11**_ and **Cu**_**12**_ catalysts. **c** ESI-MS of **Cu**_**12**_ after catalysis with NaBD_4_. Inset: the comparison of the measured (black trace) and simulated isotopic distribution patterns of [Cu_12_H_3_(Tf-dpf)_6_Cl_2_]^+^ (green, 2834.5), [Cu_12_H_3_(Tf-dpf)_6_(OAc)Cl]^+^ (blue, 2858.5), and [Cu_12_H_3_(Tf-dpf)_6_(OAc)_2_]^+^ (red, 2882.5). **d** ESI-MS of **Cu**_**11**_ after catalysis with NaBD_4_. Inset: the comparison of the measured (black trace) and simulated (red trace) isotopic distribution patterns of [Cu_11_HD_2_(Tf-dpf)_5_(OAc)_2_ + Na]^+^ (2844.6) and [Cu_11_HD_2_(Tf-dpf)_5_(OAc)]^+^ (2762.6).

The reduction process monitored by measuring the intensity change of 400 nm peak (4-NP) in UV/vis absorption spectroscopy. As the catalytic reaction proceeded in the presence of **Cu**_**11**_, the intensity of 400 nm peak decreased rapidly and disappeared within 10 min (Fig. 7a), indicating the complete conversion of 4-NP to 4-AP (*λ*_max_ = 295 nm in water). In comparison, only 5% 4-NP could be reduced to 4-AP with equivalent **Cu**_**12**_ catalyst even when the time was extended to 30 min, and the completion of reduction of 4-NP to 4-AP needed 10 h (Supplementary Fig. 12). It is quite interesting that two copper hydride clusters with similar structures show distinctly different activity in the hydrogenation reaction (Fig. 7b), which prompts us to pay efforts in mechanism study in terms of the role of hydrides.

Three major steps were generally thought to be involved in transition metal-catalyzed reduction of 4-NP to 4-AP^41,44^, and the formation of [M]–H species as well as the B–H bond cleavage was considered to be the rate-determining step. Therefore, we carried out an experiment using **Cu**_**11**_ and **Cu**_**12**_ as the catalysts for the reduction of 4-NP to 4-AP with NaBD_4_ in place of NaBH_4_. In the cases of **Cu**_**12**_, no peak belongs to deuterated cluster was found in the ESI-MS spectrum after catalysis with NaBD_4_ (Fig. 7c), which indicates that the encapsulated *μ*_6_-H of **Cu**_**12**_ were shielded from interaction with substrates. Therefore, **Cu**_**12**_ showed very low catalytic activity. On the contrary, the ESI-MS of **Cu**_**11**_ after catalysis with NaBD_4_ showed new peaks at 2762.6 and 2844.6 in addition to the expected peak of 2484.6 ([Cu_11_H_3_(Tf-dpf)_5_(OAc)_2_]^+^) (Fig. 7d). These two new peaks could be attributed to [Cu_11_HD_2_(Tf-dpf)_6_(OAc)]^+^ and [Cu_11_HD_2_(Tf-dpf)_6_(OAc)_2_ + Na]^+^, respectively, which indicates that hydrides in **Cu**_**11**_ were replaced by D atoms from NaBD_4_, i.e., the *μ*_5_-H species of **Cu**_**11**_ were involved in the catalytic cycle. These facts reveal that the high catalytic activity of **Cu**_**11**_ is related to the formation of *μ*_5_-H species on the cluster. Moreover, it is noted that **Cu**_**11**_ is relatively robust and can be re-used after centrifugation. Even after seven cycles, **Cu**_**11**_ retains its high activity (Supplementary Table 3). Previously reported copper hydride clusters including Stryker’s reagent are usually moisture- and air-sensitive. Other copper hydride clusters such as [Cu_3_H(dppm)_3_(OAc)_2_]^45,46^ and [Cu_8_H_6_(dppy)_6_](OTf)_2_^7^ are stable in solution for less than 3 days. **Cu**_**11**_ and **Cu**_**12**_ are stable in CH_2_Cl_2_ for at least 2 weeks, their good stability makes them promising copper hydride catalysts for various applications.

Overall, **Cu**_**11**_ and **Cu**_**12**_ present a pair of valuable copper hydride clusters for correlating the structures and properties. They have identical amidinate ligands, similar metal atom arrangement, but different hydride location and distinct catalytic performance, which demonstrates the importance of the location of hydrides for efficient hydrogenation catalysis^45^. This information will be instructive in the design, synthesis and selection high performance hydrogenation catalysts.

## Discussion

In summary, we have synthesized two stable copper hydride clusters **Cu**_**11**_ and **Cu**_**12**_ with the flexible amidinate ligand Tf-dpf. Because the multidentate amine ligand Tf-dpf has a negative charge and four binding N donors, it could provide strong binding to metal centers. Such a strong protection of ligand shell favors the high stability of copper hydride clusters. The compositions of these two title clusters have only one copper atom difference, but their structures and hydride positions are different. **Cu**_**11**_ and **Cu**_**12**_ show totally distinct catalytic performance in the reduction of 4-NP to 4-AP. **Cu**_**11**_ bearing *μ*_5_-H is very active while **Cu**_**12**_ with *μ*_6_-H display very low activity. Deuterated catalytic experiments prove that the hydrides at different location play key a role in the catalytic cycles. This work presents valuable information for understanding the hydrogenation process of copper hydride catalysts at atomic level, which helps optimize the design and synthesis of stable and active copper hydride catalysts.

## Methods

### Chemicals and materials

5-(trifluoromethyl)-2-aminopyridine (97%) was purchased from Meryer. Triethyl orthoformate (TEOF, 99%) and Et_3_N (99%) were purchased from Aladdin. H_2_SiPh_2_ was purchased from Bidepharm, China. CuOAc (93%) was purchased from TCI. Other reagents employed were purchased from Sinopharm Chemical Reagent Co. Ltd. (Shanghai, China). All chemical reagents employed were used without further purification.

### Synthesis of N,N′-Di(5-trifluoromethyl-2-pyridyl)formamidine (HTf-dpf)

Excess TEOF (1.8571 g, 17.5 mmol) was added to of 5-(trifluoromethyl)-2-aminopyridine (3.2422 g, 20.0 mmol) and heated at 120 °C under a low nitrogen stream for 5 h. The excessed TEOF and ethanol formed during the reaction were distilled off, and the product was recrystallized from petroleum ether/methanol (5:1). Yield: 2.97 g, 89%.

Anal. UV–Vis (*λ*, nm): 231; 267; 323. ^1^H NMR (400 MHz, DMSO-*d*_6_, *δ*, ppm): 11.41 (s, 1H, –CH–), 9.77 (s, 1H, NH), 8.70 (s, 2H, py), 8.11–8.08 (dd, 2H, py), 7.22 (s, 2H, py). ^19^F NMR (400 MHz, DMSO-*d*_6_, *δ*, ppm): −60.21. ^13^C NMR (400 MHz, DMSO-*d*_6_, *δ*, ppm): 151.70, 146.17, 136.20, 128.70, 126.00, 123.31, 120.61.

### Synthesis of [Cu_12_(Tf-dpf)_6_(OAc)_2_H_3_]·OAc (Cu_12_)

In total, 3 ml CH_2_Cl_2_/CH_3_OH (v:v = 2:1) mixture of Cu(OAc) (24 mg, 0.2 mmol), HTf-dpf (0.1 mmol, 33.4 mg), and excess Et_3_N (20 ul) was stirred for 5 min first, then H_2_SiPh_2_ (0.1 mmol, 18 ul) was added. The solution color changed from green to yellow in 10 min. The mixture was stirred for 3 h and evaporated to dryness to give a yellow solid, which was washed with n-hexane (3 × 2 ml), then dissolved in 4 ml CH_2_Cl_2_/CH_3_OH (v:v = 3:1). The resulted solution was centrifuged for 2 min at 9000 r/min, and the yellow supernatant was collected and subjected to diffusion with n-hexane to afford light yellow crystals after 2 days in 27.4 mg, 56% yield (based on Cu).

Anal. UV–Vis (*λ*, nm): 238; 288; 341. ESI-MS (CH_3_OH): 2882.51 ([Cu_12_(Tf-dpf)_6_(OAc)_2_H_3_]^+^). ^1^H NMR (400 MHz, CD_3_OD, *δ*, ppm): 8.95 (m, 6H, –CH–), 8.55 (m, 12H, py), 7.59–7.57 (m, 12H, py), 7.23–7.20 (m, 12H, py), 7.16 (s, 1H, hydride), 5.64 (s, 2H, hydride), 3.59 (s, 3H, –CH_3_), 2.68 (s, 6H, –CH_3_). ^19^F NMR (400 MHz, CD_3_OD, *δ*, ppm): −63.88.

### Synthesis of Cu_11_(Tf-dpf)_6_(OAc)_2_H_3_ (Cu_11_)

In total, 3 ml CH_2_Cl_2_/DMSO (v:v = 5:1) mixture of Cu(OAc) (24 mg, 0.2 mmol), HTf-dpf (0.1 mmol, 33.4 mg), and excess Et_3_N (20 ul) was stirred for 5 min first, then H_2_SiPh_2_ (0.1 mmol, 18 ul) was added. The solution color changed from green to yellow in 10 min. The mixture was stirred for 3 h and evaporated to remove the CH_2_Cl_2_ solvent. The crude product was washed by 5 ml CH_2_Cl_2_/n-hexane (v:v = 1:4) for three times, then dissolved in 4 ml CH_2_Cl_2_. The resulted solution was centrifuged for 2 min at 9000 r/min, and the orange supernatant was collected and subjected to diffusion with n-hexane to afford light orange crystals after 2 days in 24.2 mg, 47% yield (based on Cu).

Anal. UV–Vis (*λ*, nm): 240; 287; 338. ESI-MS (CH_3_OH): 2819.64 ([Cu_11_(Tf–dpf)_6_(OAc)_2_H_3_]^+^) and 2484.56 ([Cu_11_(Tf-dpf)_5_(OAc)_2_H_3_]^+^). ^1^H NMR (400 MHz, CD_3_OD, *δ*, ppm): 8.98–8.54 (m, 12H, –CH– and py), 7.59–7.50 (m, 12H, py), 7.21–7.17 (m, 12H, py), 3.15 (s, 1H, hydride), 2.60 (s, 6H, –CH_3_), 2.34 (s, 2H, hydride). ^19^F NMR (400 MHz, CD_3_OD, *δ*, ppm): −63.28.

### Catalytic reduction of 4-nitrophenol

The water solution of 4-NP (1 ml, 20 mM), **Cu**_**11**_ or **Cu**_**12**_ (1 mg) was mixed, and the mixture was stirred for 10 min at room temperature. Time-resolved UV–vis spectra were taken immediately after the addition of NaBH_4_ solid (50 mg, 1.3 mmol). The progress of the reaction was tracked by monitoring the change in intensity of 4-NP peak at 400 nm as a function of time. After reaction of **Cu**_**11**_, the reaction solution was centrifuged, and the catalysts was washed with 3 ml H_2_O for three times. Then the catalysts solid was dried under reduced pressure and re-used as fresh.

### Neural network prediction of hydride sites

We employed the recently developed deep-learning model to predict hydride sites in our clusters. The model was based on CNN and trained on Cu-H clusters with hydride sites determined by neutron diffraction. This model takes as input the heavy-atom coordinates of a cluster from the single-crystal X-ray diffraction and then outputs the occupancy for each possible hydride site in the cluster. The training data are based on 23 different copper hydride clusters from the Cambridge Structural Database whose hydride locations have been determined by neutron diffraction. The 23 structures were further chunked into 674 boxes of possible hydride sites that were used for training of CNN. The details of the CNN and its architecture can be found in the previous work^39,40^ and their Supporting Information. The trained CNN can classify a possible site for hydride in a given cluster with accuracy higher than 94%. In the present work, the X-ray structures of the **Cu**_**11**_ and **Cu**_**12**_ clusters (namely, coordinates of Cu, C, N, F, and O in the cluster) were used as input into the machine-learning model which then predicted hydride occupancies and ranked the hydride sites. Since there are only three hydrides in the **Cu**_**11**_ and **Cu**_**12**_ clusters, one can simply pick the top-ranked sites and examine the top three by inspection, followed by DFT geometry optimization for confirmation using the VASP code.

### Physical measurements

UV–Vis absorption spectra was recorded on cary5000. Mass spectra were recorded on a high-resolution Fourier transform ICR spectrometer with an electrospray ionization source in positive mode. Nuclear magnetic resonance data were recorded on a Bruker Avance II spectrometer (500 MHz).

### X-ray crystallography

Intensity data of compounds Cu_11_ and Cu_12_ were collected on an Agilent SuperNova Dual system (Cu Kα) at 173 K. Absorption corrections were applied by using the program CrysAlis (multi-scan). The structures of Cu_11_ and Cu_12_ were solved by direct methods. Non-hydrogen atoms except solvent molecules and counteranions were refined anisotropically by least-squares on *F*^2^ using the SHELXTL program. For Cu_12_, the -CF_3_ groups (F7–F9, F22–F24) were disordered over two sites with an occupancy factor of 0.5/0.5. SQUEEZE routine in PLATON was employed in the structural refinements due to large solvent voids. In addition, isor and rigu constraints have been applied due to geometric requirements of the ligands.

### Computational methods

DFT calculations were performed with the quantum chemistry program Turbomole V7.1^47^. The Def2-SV(P) basis sets^48^ were used for C, N, O, H, F. The Def2-TZVP basis sets^49^ were used for Cu. Geometry optimization was done with the functional of Perdew, Burke and Ernzerhof^50^.

## Supplementary information

Supplementary Information

## Data Availability

The data that support the findings of this study are available from the corresponding author upon reasonable request. The X-ray crystallographic coordinates for structures reported in this article (see Supplementary Table 1) have been deposited at the Cambridge Crystallographic Data Centre (CCDC) under deposition numbers CCDC 2100815 (**Cu**_**11**_) and CCDC 2100816 (**Cu**_**12**_). These data can be obtained free of charge from the Cambridge Crystallographic Data Centre via http://www.ccdc.cam.ac.uk/data_request/cif.

## References

[1] Jordan AJ, Lalic G, Sadighi JP (2016). Coinage metal hydrides: synthesis, characterization, and reactivity. Chem. Rev..

[2] Liu X, Astruc D (2018). Atomically precise copper nanoclusters and their applications. Coord. Chem. Rev..

[3] Sun C (2021). Hydrido-coinage-metal clusters: rational design, synthetic protocols and structural characteristics. Coord. Chem. Rev..

[4] Zhu S, Niljianskul N, Buchwald SL (2016). A direct approach to amines with remote stereocentres by enantioselective CuH-catalysed reductive relay hydroamination. Nat. Chem..

[5] Wang YM, Buchwald SL (2016). Enantioselective CuH-catalyzed hydroallylation of vinylarenes. J. Am. Chem. Soc..

[6] Deutsch C, Krause N (2008). CuH-catalyzed reactions. Chem. Rev..

[7] Yuan S-F (2021). A stable well-defined copper hydride cluster consolidated with hemilabile phosphines. Chem. Commun..

[8] Dhayal RS, van Zyl WE, Liu CW (2016). Polyhydrido copper clusters: synthetic advances, structural diversity, and nanocluster-to-nanoparticle conversion. Acc. Chem. Res..

[9] Chakrahari KK (2016). [Cu_13_{S_2_CN^n^Bu_2_}_6_(acetylide)_4_]^+^: a two-electron superatom. Angew. Chem. Int. Ed..

[10] Dhayal RS (2015). Diselenophosphate-induced conversion of an achiral [Cu_20_H_11_{S_2_P(OiPr)_2_}_9_] into a chiral [Cu_20_H_11_{Se_2_P(OiPr)_2_}_9_] polyhydrido nanocluster. Angew. Chem. Int. Ed..

[11] Edwards AJ (2014). Chinese puzzle molecule: a 15 hydride, 28 copper atom nanoball. Angew. Chem. Int. Ed..

[12] Huang R-W (2020). [Cu_23_(PhSe)_16_(Ph_3_P)_8_(H)_6_]·BF_4_: atomic-level insights into cuboidal polyhydrido copper nanoclusters and their quasi-simple cubic self-assembly. ACS Mater. Lett..

[13] Lee S (2020). [Cu_32_(PET)_24_H_8_Cl_2_](PPh_4_)_2_: a copper hydride nanocluster with a bisquare antiprismatic core. J. Am. Chem. Soc..

[14] Nakamae K, Nakajima T, Ura Y, Kitagawa Y, Tanase T (2020). Facially dispersed polyhydride Cu_9_ and Cu_16_ clusters comprising apex-truncated supertetrahedral and square-face-capped cuboctahedral copper frameworks. Angew. Chem. Int. Ed..

[15] Yuan P (2019). Ether-soluble Cu_53_ nanoclusters as an effective precursor of high-quality CuI films for optoelectronic applications. Angew. Chem. Int. Ed..

[16] Tang Q (2017). Lattice-hydride mechanism in electrocatalytic CO_2_ reduction by structurally precise copper-hydride nanoclusters. J. Am. Chem. Soc..

[17] Dhayal RS (2013). A nanospheric polyhydrido copper cluster of elongated triangular orthobicupola array: liberation of H_2_ from solar energy. J. Am. Chem. Soc..

[18] Barik SK (2020). Polyhydrido copper nanoclusters with a hollow icosahedral core: [Cu_30_H_18_{E_2_P(OR)_2_}_12_] (E=S or Se; R = *n*Pr, *i*Pr or *i*Bu). Chem. Eur. J..

[19] Chakrahari, K. K. et al. Isolation and structural elucidation of 15-nuclear copper dihydride clusters: an intermediate in the formation of a two-electron copper superatom. *Small* **17**, 2002544 (2020). 10.1002/smll.202002544 33113288

[20] Nasaruddin RR, Chen T, Yan N, Xie J (2018). Roles of thiolate ligands in the synthesis, properties and catalytic application of gold nanoclusters. Coord. Chem. Rev..

[21] Kang X. & Zhu, M. Z. Metal nanoclusters stabilized by selenol ligands. *Small* **15**, 1902703 (2019). 10.1002/smll.201902703 31482648

[22] Wan X-K, Wang J-Q, Nan Z-A, Wang Q-M (2017). Ligand effects in catalysis by atomically precise gold nanoclusters. Sci. Adv..

[23] Kurashige W, Yamaguchi M, Nobusada K, Negishi Y (2012). Ligand-induced stability of gold nanoclusters: thiolate versus selenolate. J. Phys. Chem. Lett..

[24] Guan ZJ (2016). Thiacalix [4] arene: new protection for metal nanoclusters. Sci. Adv..

[25] Lei Z, Wan X-K, Yuan S-F, Wang J-Q, Wang Q-M (2017). Alkynyl-protected gold and gold-silver nanoclusters. Dalton. Trans..

[26] Liu KG, Gao XM, Liu T, Hu ML, Jiang DE (2020). All-carboxylate-protected superatomic silver nanocluster with an unprecedented rhombohedral Ag_8_ core. J. Am. Chem. Soc..

[27] Liu W-D, Wang J-Q, Yuan S-F, Chen X, Wang Q-M (2021). Chiral superatomic nanoclusters Ag_47_ induced by the ligation of amino acids. Angew. Chem. Int. Ed..

[28] Yuan S-F (2021). Robust gold nanocluster protected with amidinates for electrocatalytic CO_2_ reduction. Angew. Chem. Int. Ed..

[29] Yuan S-F, Lei Z, Guan Z-J, Wang QM (2021). Atomically precise preorganization of open metal sites on gold nanoclusters with high catalytic performance. Angew. Chem. Int. Ed..

[30] Yuan S-F, Guan Z-J, Liu W-D, Wang Q-M (2019). Solvent-triggered reversible interconversion of all-nitrogen-donor-protected silver nanoclusters and their responsive optical properties. Nat. Commun..

[31] Kounalis E, Lutz M, Broere DLJ (2019). Cooperative H_2_ activation on dicopper(I) facilitated by reversible dearomatization of an “Expanded PNNP Pincer” ligand. Chem. Eur. J..

[32] Desnoyer AN, Nicolay A, Ziegler MS, Torquato NA, Tilley TD (2020). A dicopper platform that stabilizes the formation of pentanuclear coinage metal hydride complexes. Angew. Chem. Int. Ed..

[33] Kombe H, Limbach HH, Böhme F, Kunert C (2002). NMR studies of the tautomerism of Cyclo-tris(4-R-2,6-pyridylformamidine) in solution and in the solid state. J. Am. Chem. Soc..

[34] Nguyen T-AD (2016). Ligand-exchange-induced growth of an atomically precise Cu_29_ nanocluster from a smaller cluster. Chem. Mat..

[35] Guo QL (2021). Observation of a bcc-like framework in polyhydrido copper nanoclusters. Nanoscale.

[36] Liao PK (2011). A copper(I) homocubane collapses to a tetracapped tetrahedron upon hydride insertion. Inorg. Chem..

[37] Liao, P. K. et al. Hydrido copper clusters supported by dithiocarbamates: oxidative hydride removal and neutron diffraction analysis of [Cu_7_(H){S_2_C(aza-15-crown-5)}_6_]. *Inorg. Chem.* **51**, 6577–6591 (2012). 10.1021/ic300135w 22663192

[38] Kohn RD, Pan Z, Mahon MF, Kociok-Kohn G (2003). Trimethyltriazacyclohexane as bridging ligand for triangular Cu_3_ units and C-H hydride abstraction into a Cu_6_ cluster. Chem. Commun..

[39] Wang S, Wu ZL, Dai S, Jiang DE (2021). Deep learning accelerated determination of hydride locations in metal nanoclusters. Angew. Chem. Int. Ed..

[40] Wang S, Liu T, Jiang DE (2021). Locating hydrides in ligand-protected copper nanoclusters by deep learning. Acs Appl. Mater. Int..

[41] Zhao P, Feng X, Huang D, Yang G, Astruc D (2015). Basic concepts and recent advances in nitrophenol reduction by gold- and other transition metal nanoparticles. Coord. Chem. Rev..

[42] Tamiolakis I, Fountoulaki S, Vordos N, Lykakis IN, Armatas GS (2013). Mesoporous Au–TiO_2_ nanoparticle assemblies as efficient catalysts for the chemoselective reduction of nitro compounds. J. Mater. Chem. A.

[43] Wunder S, Lu Y, Albrecht M, Ballauff M (2011). Catalytic activity of faceted gold nanoparticles studied by a model reaction: evidence for substrate-induced surface restructuring. ACS Catal..

[44] Sun C (2019). Atomically precise, thiolated copper–hydride nanoclusters as single-site hydrogenation catalysts for ketones in mild conditions. ACS Nano.

[45] Fountoulaki S (2014). Mechanistic studies of the reduction of nitroarenes by NaBH_4_ or hydrosilanes catalyzed by supported gold nanoparticles. ACS Catal..

[46] Cook AW, Nguyen TD, Buratto WR, Wu G, Hayton TW (2016). Synthesis, characterization, and reactivity of the group 11 hydrido clusters [Ag_6_H_4_(dppm)_4_(OAc)_2_] and [Cu_3_H(dppm)_3_(OAc)_2_]. Inorg. Chem..

[47] Furche F (2014). Turbomole. WIREs Comput. Mol. Sci..

[48] Weigend F, Haser M, Patzelt H, Ahlrichs R (1998). RI-MP2: optimized auxiliary basis sets and demonstration of efficiency. Chem. Phys. Lett..

[49] Weigenda F, Ahlrichs R (2005). Balanced basis sets of split valence, triple zeta valence and quadruple zeta valence quality for H to Rn: Design and assessment of accuracy. Phys. Chem. Chem. Phys..

[50] Ahlrichs R, Bar M, Haser M, Horn H, Kolmel C (1989). Electronic structure calculations on workstation computers: the program system turbomole. Chem. Phys. Lett..

