# Supplementary material for: Structural transformation and catalytic hydrogenation activity of amidinate-protected copper hydride clusters
Source: Nat Commun. 2022 Apr 19;13:2082. doi: 10.1038/s41467-022-29819-y (PMC9018778; doi:10.1038/s41467-022-29819-y)
Supplement: Supplementary file 1 — Supplementary Information [file 41467_2022_29819_MOESM1_ESM.pdf]

Supplementary Information

Structural Transformation and Catalytic Hydrogenation Activity of Amidinate-Protected Copper Hydride Clusters

Chun-Yu Liu,[1] Shang-Fu Yuan,[1] Song Wang,[2] Zong-Jie Guan,[1] De-en Jiang,[2] and Quan-Ming Wang*,[1]

[1]Department of Chemistry, Key Laboratory of Organic Optoelectronics and Molecular Engineering of the Ministry of Education, Tsinghua University, Beijing 10084, PR China

[2]Department of Chemistry, University of California, Riverside, California 92521, United States

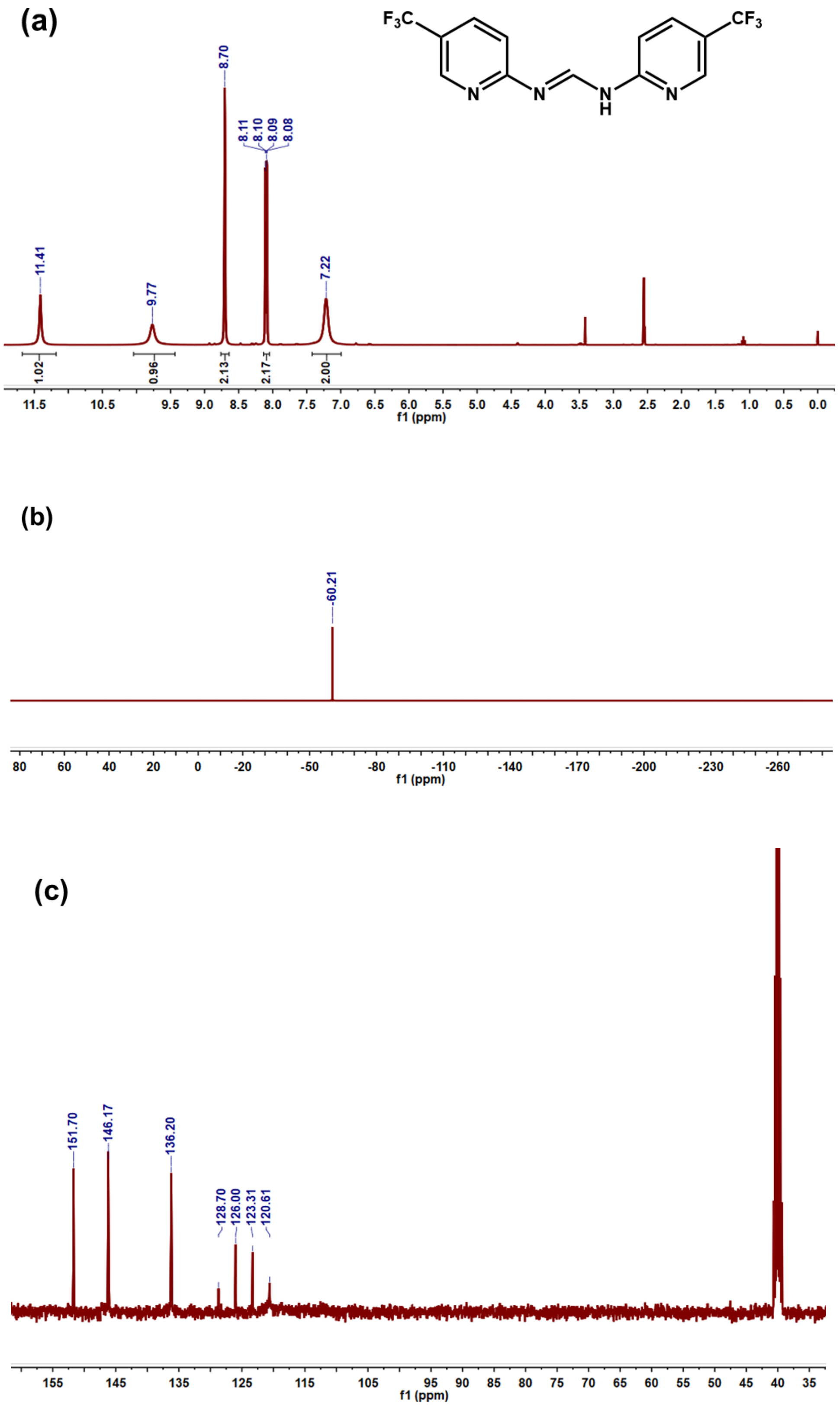

Supplementary Figure 1. The NMR spectra of HTf-dpf. The ^{1}H NMR (**a**), ^{19}F NMR (**b**) and ^{13}C NMR (**c**) spectrums for **HTf-dpf** in DMSO-d_6.

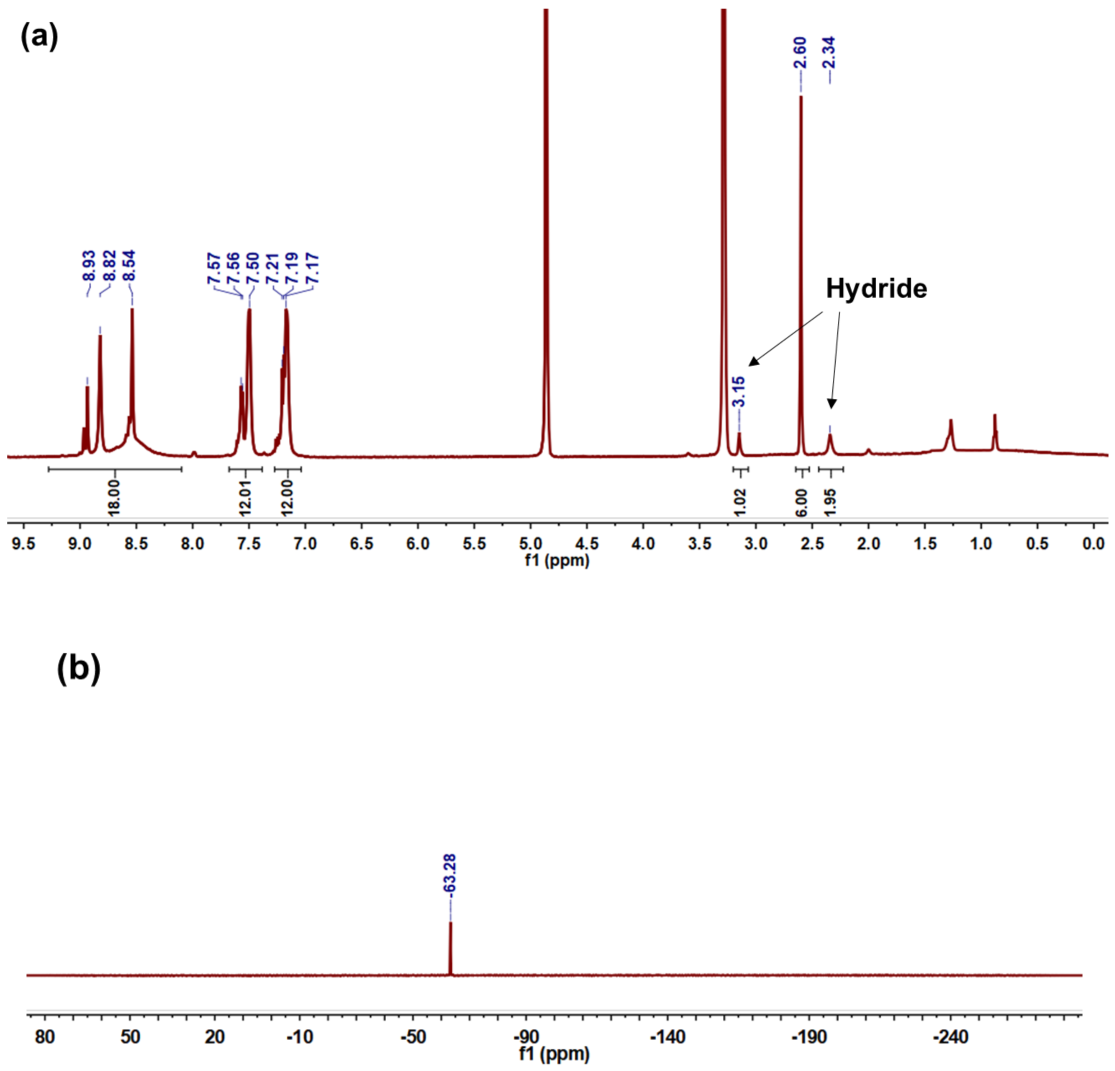

Supplementary Figure 2. The NMR spectra of Cu_{11}. The ^{1}H NMR (**a**) and ^{19}F NMR (b) spectrums for **Cu_{11}** in CD_3OD.

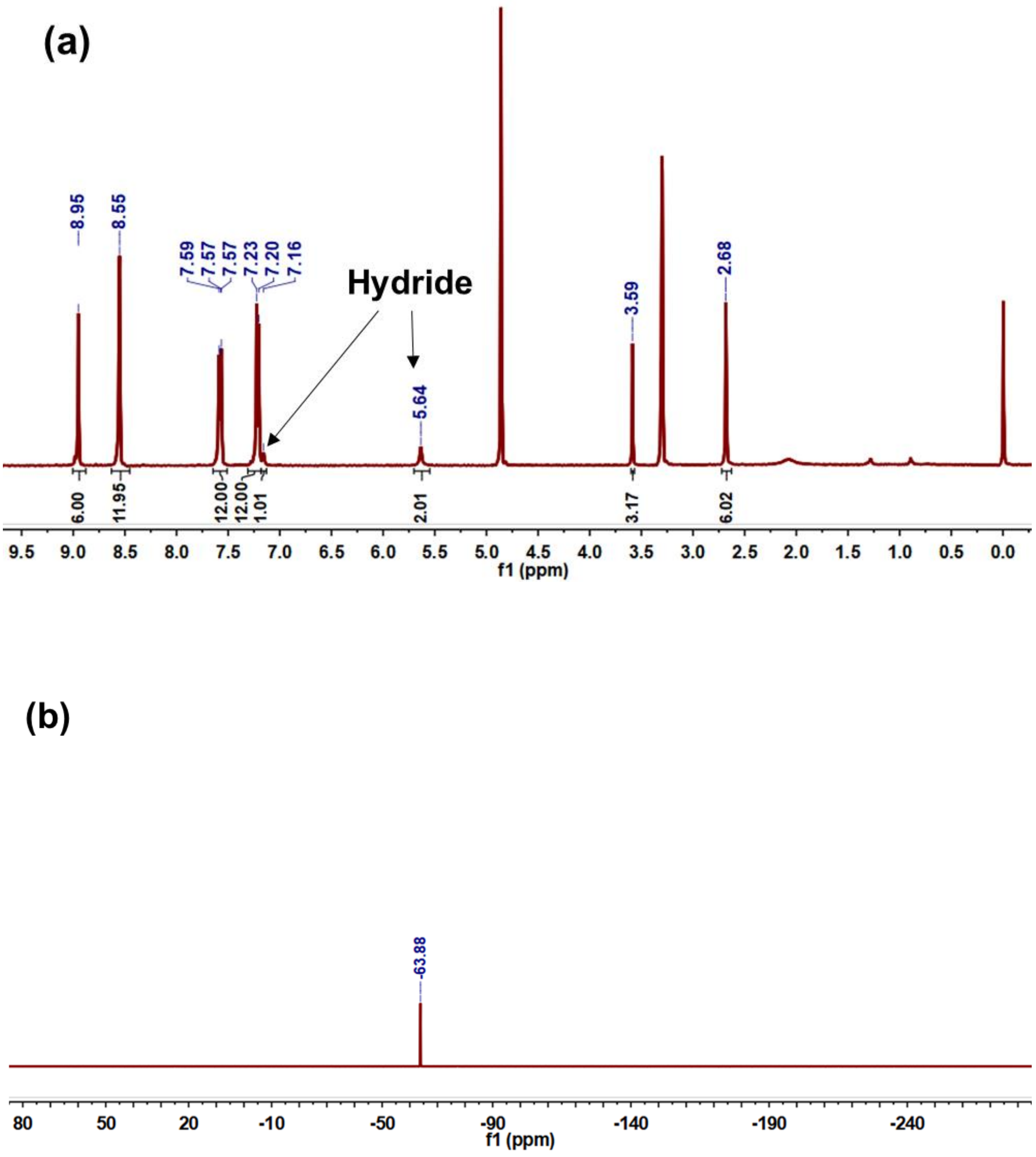

Supplementary Figure 3. The NMR spectra of Cu_{12}. The ^{1}H NMR (**a**) and ^{19}F NMR (**b**) spectrums for **Cu_{12}** in CD_3OD.

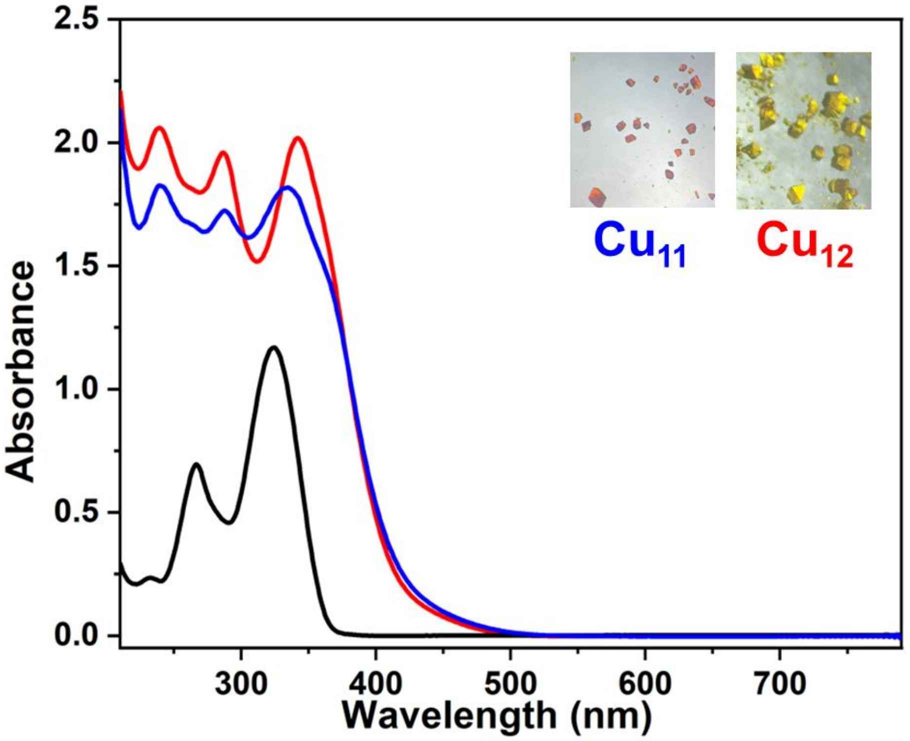

Supplementary Figure 4. UV-vis absorption characteristic curves of the ligand and the clusters in CH_3OH. HTf-dpf (black), **Cu_{11}** (blue), and **Cu_{12}** (red). Inset: the photographs of **Cu_{11}** and **Cu_{12}** crystals.

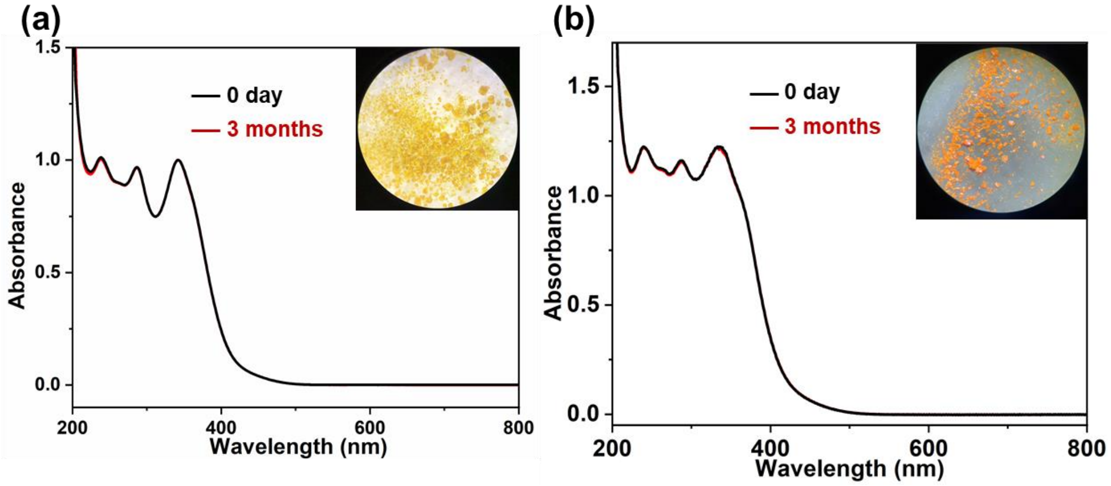

Supplementary Figure 5. Stability of Cu_{11} and Cu_{12} in the solid state. UV-vis absorption spectra of **Cu_{11}** (**a**) and **Cu_{12}** (**b**) (solid powder was dissolved in MeOH at different times). Inset is a picture of solid powder of **Cu_{11}** (**a**) and **Cu_{12}** (**b**), respectively.

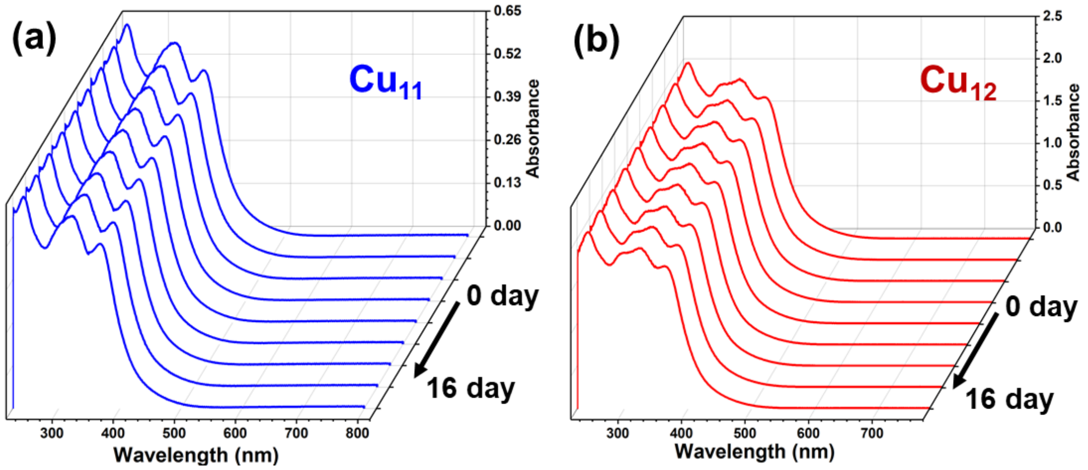

Supplementary Figure 6. Stability of Cu_{11} and Cu_{12} in CH_2Cl_2. Time dependent UV–vis absorption spectra of **Cu_{11}** (**a**) and **Cu_{12}** (**b**) in CH_2Cl_2 solution at room temperature.

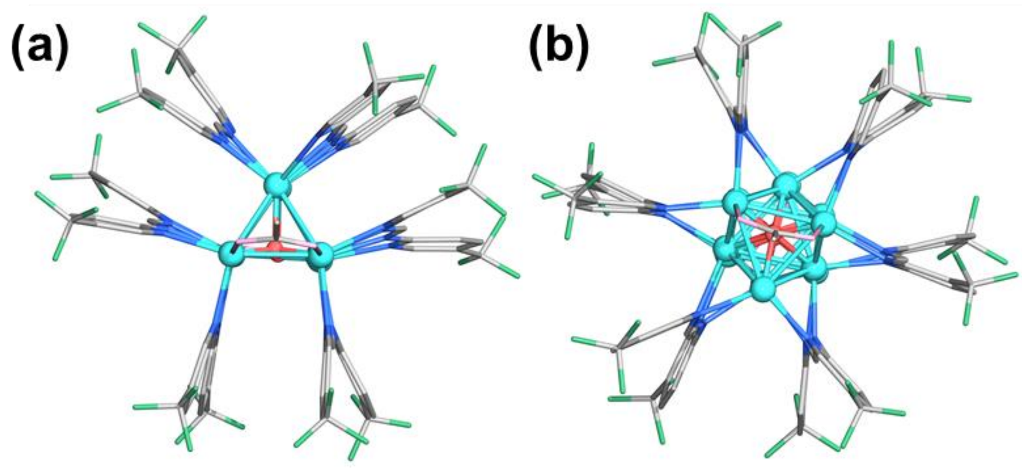

Supplementary Figure 7. Molecular structures of Cu_{11} and Cu_{12}. Side view of $\mathbf{Cu_{11}}$ (**a**) and $\mathbf{Cu_{12}}$ (**b**). Color legend: light blue, Cu; green, F; blue, N; pink, O; gray, C; red, H.

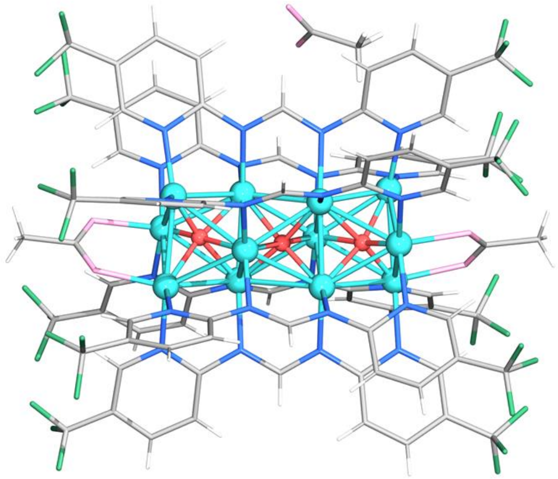

Supplementary Figure 8. The $[Cu_{12}(Tf\text{-}dpf)_6(OAc)_2H_3]^+$ cationic cluster and OAc^- counteranion in **Cu_{12}**. Color legend: light blue, Cu; green, F; blue, N; pink, O; gray, C; white, H; red, hydrides.

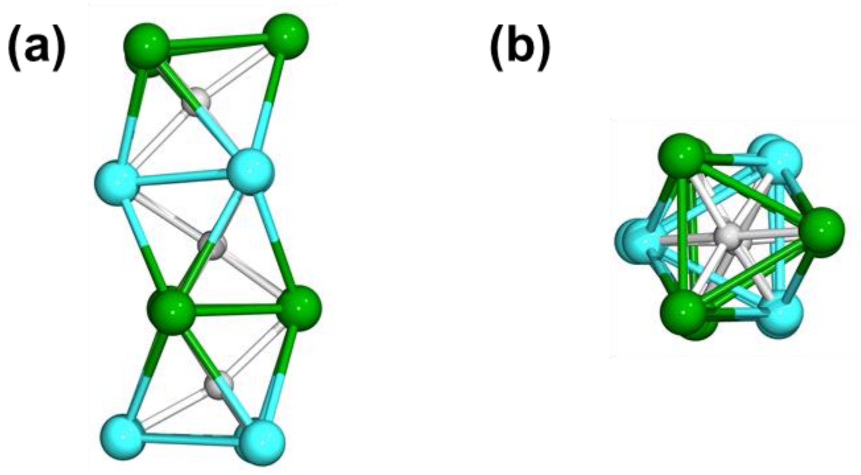

Supplementary Figure 9. The hcp structure in Cu_{12}. Side view (**a**) and top view (**b**) of the hcp structure of $Cu_{12}(\mu_6\text{-}H)_3$ core in **Cu_{12}**.

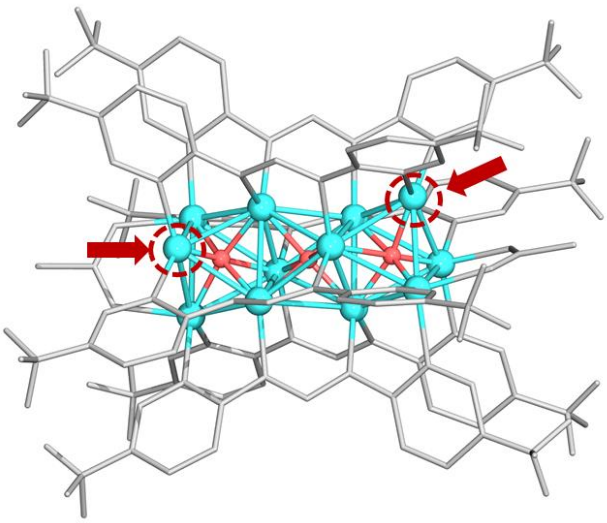

Supplementary Figure 10. The unsaturated copper atom of $Cu_{12}(\mu_6\text{-}H)_3$ core in **Cu_{12}**.

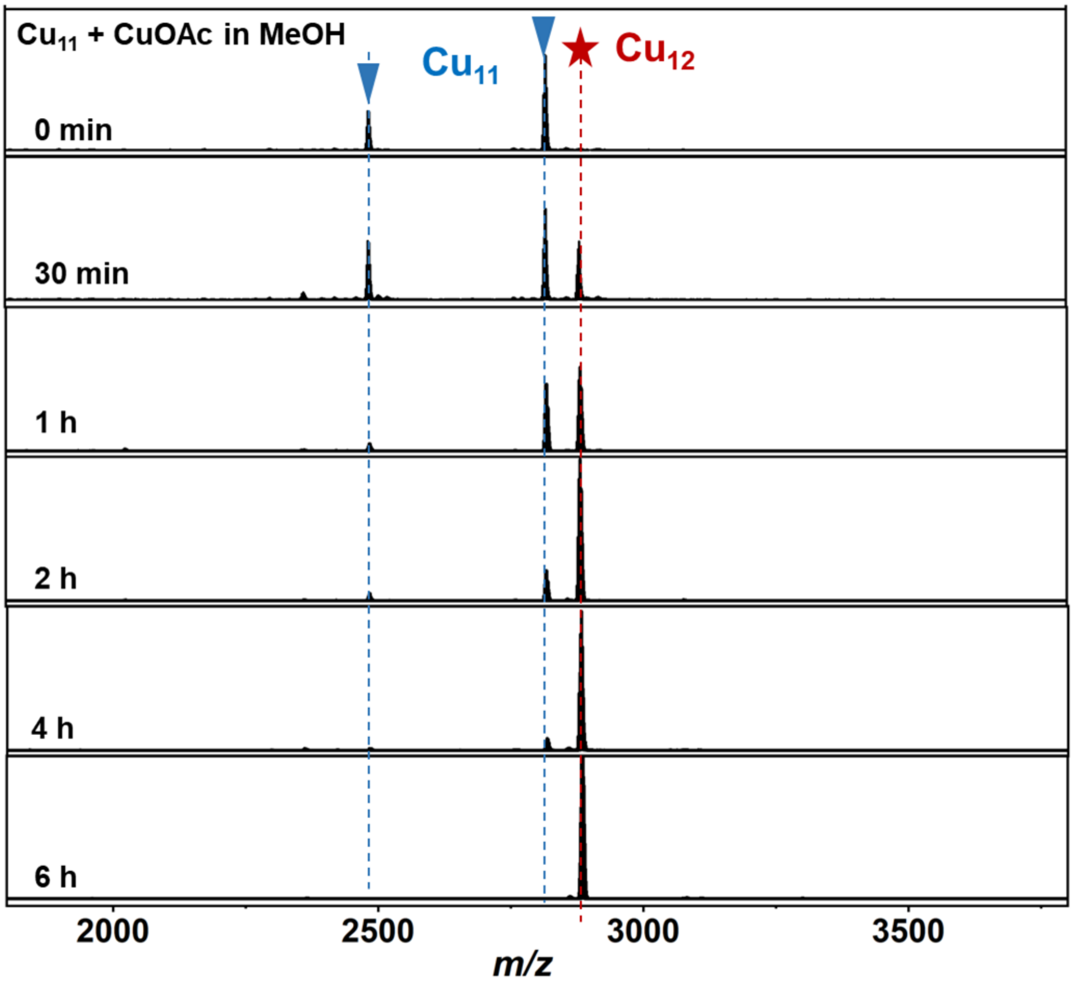

Supplementary Figure 11. Conversion of Cu_{11} to Cu_{12}. Time-dependent ESI-MS spectrum in the transformation from **Cu_{11}** to **Cu_{12}**.

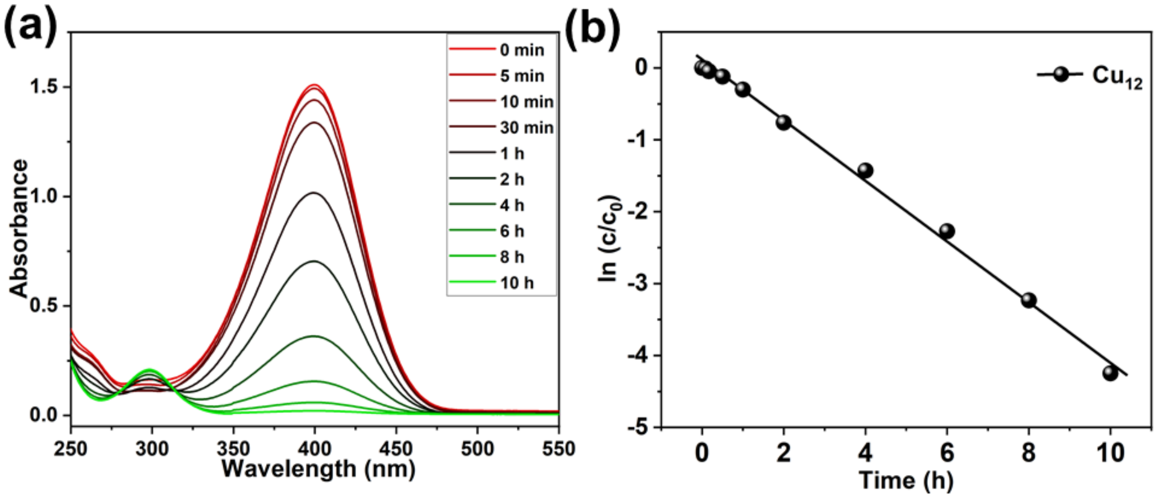

Supplementary Figure 12. Catalytic performance of Cu12. (a) UV-Vis spectra showing gradual reduction of 4-NP catalysed by **Cu12**. (b) Plot of −ln(c/c_0) *vs*. reaction time during the reduction of 4-NP with **Cu12** catalysts.

Supplementary Table 1. Crystal data and structure refinement for **Cu_{11}** and **Cu_{12}**.

	**Cu_{11}**	**Cu_{12}**
Empirical formula	$C_{82}H_{51}Cu_{11}F_{36}N_{24}O_4$	$C_{84}H_{54}Cu_{12}F_{36}N_{24}O_6$
Formula weight	2819.41	2941.99
Temperature/K	173.00(10)	173.00(10)
Crystal system	monoclinic	triclinic
Space group	$P2_1/c$	$P\bar{1}$
a/Å	23.9611(2)	14.1248(3)
b/Å	27.7077(3)	14.9567(3)
c/Å	17.33354(15)	28.5416(5)
α/°	90	83.4640(10)
β/°	98.2556(9)	82.997(2)
γ/°	90	75.343(2)
Volume/Å^3	11388.63(18)	5768.3(2)
Z	4	2
ρ_{calc}g/cm^3	1.644	1.694
μ/mm^{-1}	3.192	3.368
F(000)	5544	2892
Radiation	Cu Kα (λ = 1.54184)	Cu Kα (λ = 1.54184)
2Θ range for data collection/ °	7.39 to 147.746	7.122 to 130.354
Reflections collected	81745	36094
Independent reflections	22713 [R_{int} = 0.0428, R_{sigma} = 0.0424]	18906 [R_{int} = 0.0356, R_{sigma} = 0.0426]
Goodness-of-fit on F^2	1.113	1.035
Final R indexes [I>=2σ (I)]	R_1 = 0.0637, wR_2 = 0.1670	R_1 = 0.0624, wR_2 = 0.1669
Final R indexes [all data]	R_1 = 0.0806, wR_2 = 0.1769	R_1 = 0.0668, wR_2 = 0.1709

Supplementary Table 2. Selected structural parameters (DFT computed values) of **Cu_{11}** and **Cu_{12}**.

**Complex**	**Bond**	**Bond length (Å) from DFT**
**Cu_{11}**	Cu-H	1.743-1.915
	Cu-Cu	2.456-2.736
	Cu-N	2.053-2.133
	Cu-O	2.042-2.060
**Cu_{12}**	Cu-H	1.749-2.061
	Cu-Cu	2.527-2.753
	Cu-N	2.023-2.118
	Cu-O	2.112-2.134

Supplementary Table 3. Catalysis recycle test of **Cu_{11}**.

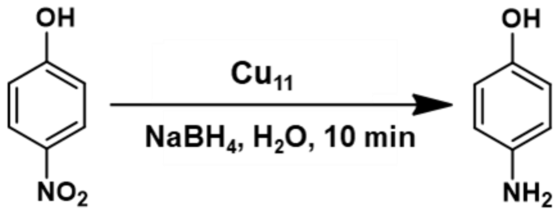

**Number of cycles**[a]	**Yield (%)**[b]
1	100
2	100
3	100
4	100
5	100
6	100
7	100

[a]Reaction condition: 4-nitrophenol (3.2 mg, 2.3×10^{-2} mmol), **Cu_{11}** (1 mg, 3.55×10^{-4} mmol), H_2O (2 mL), $NaBH_4$ (50 mg, 1.3 mmol). [b]monitored by UV-Vis absorption spectra.
